# Case report and operative management of gallbladder herniation

**DOI:** 10.1186/s12893-015-0056-7

**Published:** 2015-06-11

**Authors:** Henry To, Stephen Brough, Girish Pande

**Affiliations:** Department of Surgery, Royal Melbourne Hospital, Melbourne, Victoria Australia; Department of Surgery, Launceston General Hospital, Launceston, Tasmania Australia

**Keywords:** Gallbladder, Hernia, Incarcerated, Parastomal, Cholecystitis

## Abstract

**Background:**

Incarcerated abdominal wall hernias may contain a variety of contents, but very rarely contains the gallbladder. This rare diagnosis is often not considered and, when diagnosed, has a different management approach. The experience of the small number of case reports have yet to be collected and summarised.

**Case presentation:**

We report a presentation and management of an 85 year old Caucasian female with a gallbladder hernia into a parastomal defect, and outline the operative management.

**Conclusion:**

Incarcerated gallbladder hernia is an extremely unusual condition, best diagnosed by CT scan. Management should involve operative reduction, cholecystectomy and consideration of repair of the defect.

## Background

A hernia is a protrusion of a viscus, or part of a viscus, through the walls of its containing cavity [1]. A variety of intra-abdominal organs may be found in a hernia sac, such as omentum, small bowel, colon, bladder, appendix, stomach, ovary or fallopian tube (or a combination of these). In the era of computerised tomography (CT) radiology, the contents of abdominal wall hernias may be more readily identified [2]. There is a need to be aware of the potential contents of hernia sacs, which has implications for definitive operative management. An incarcerated gallbladder hernia is a rare condition, and a small number of case reports describe the process of diagnosis and subsequent management. This case report outlines the presentation, diagnosis and operative management of one such patient, and collates the management principles based on the few reported cases of this condition.

## Case presentation

An 85 year old Caucasian female was admitted with a four day history of increasing abdominal pain at her ileal conduit site with nausea but no vomiting. Five years prior, she was diagnosed with a high grade urothelial carcinoma, and underwent a total cystectomy with formation of a right iliac fossa ileal conduit. There was no evidence of tumour recurrence after 5 years of follow up. She did not have any other previous abdominal surgery and no significant past medical history.

**Fig. 1:**
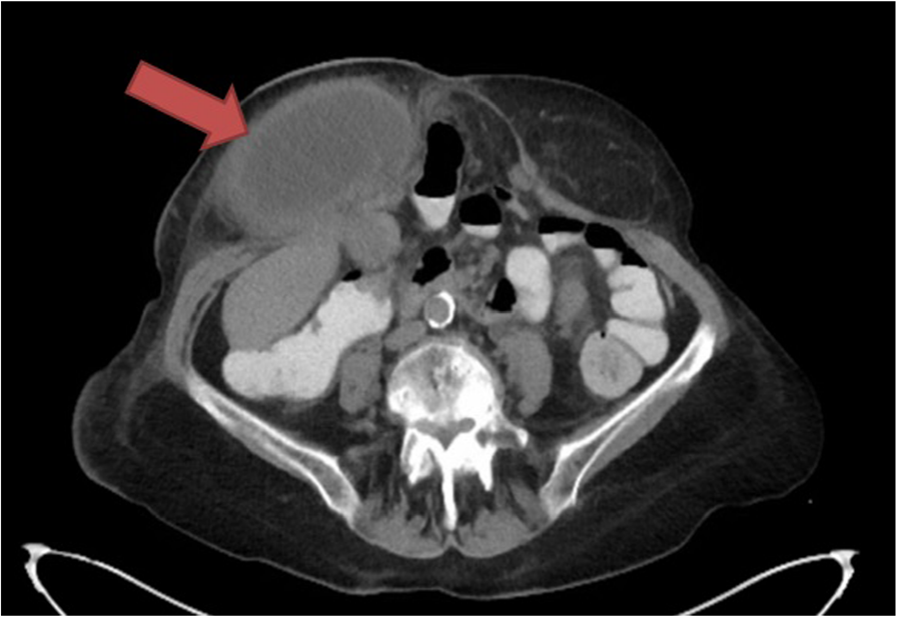
Computerised tomography appearance of gallbladder hernia (red arrow) in transverse view

At the time of her presentation, she had mild fever but did not have vomiting. She had normal bowel function and her urine output via the ileostomy was normal. Physical examination revealed a firm, irreducible mass lateral to her stomal site with overlying erythema. The stoma was healthy in appearance. There was also a reducible midline abdominal hernia. Her white cell count was elevated but all other blood tests including electrolytes, renal function, bilirubin and liver function tests were normal. A CT scan with oral gastrograffin contrast showed a midline abdominal hernia with small bowel loops, and a parastomal hernia containing an enlarged, thick-walled mass which did not contain oral contrast (Fig. 1). With close proximity to the liver and absence of the gallbladder from its anatomical position, a gallbladder hernia was suspected. The alternative diagnosis of herniation of small bowel was considered, but with lack of oral contrast within the hernia, it was considered less likely.

**Fig. 2:**
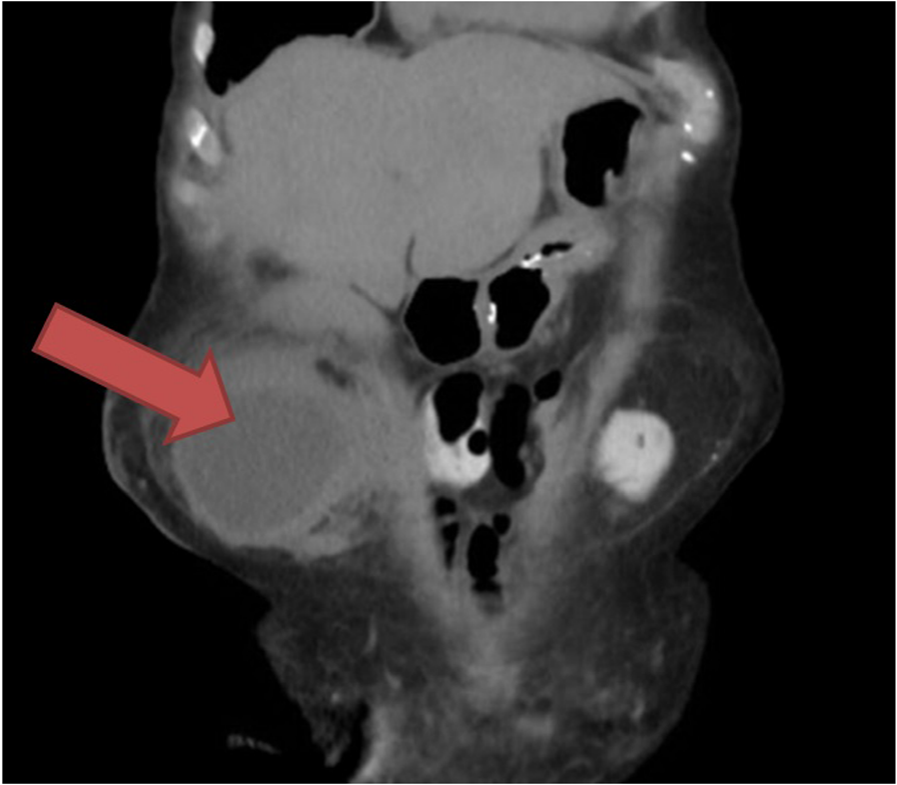
CT appearance in coronal views

**Fig. 3:**
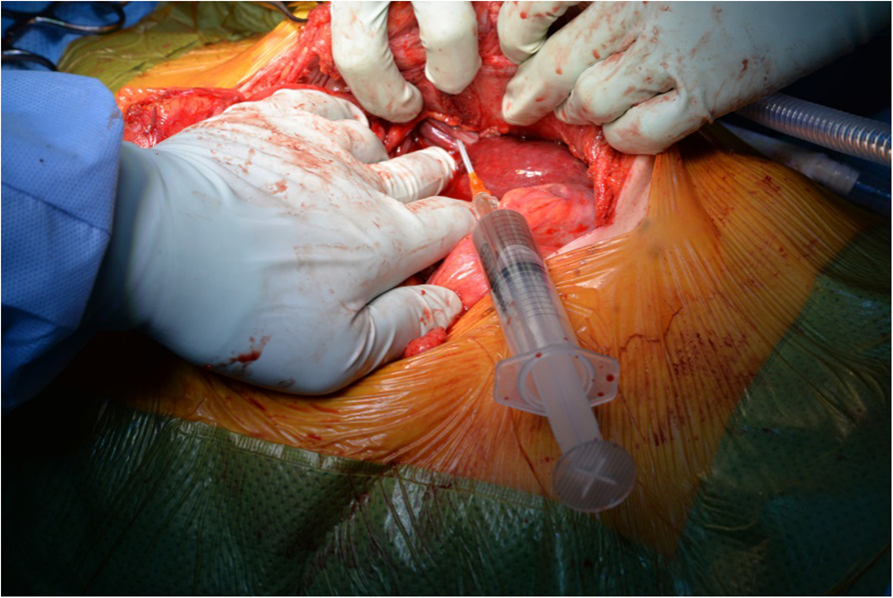
Identification of parastomal defect at laparotomy demonstrating the gallbladder neck

**Fig. 4:**
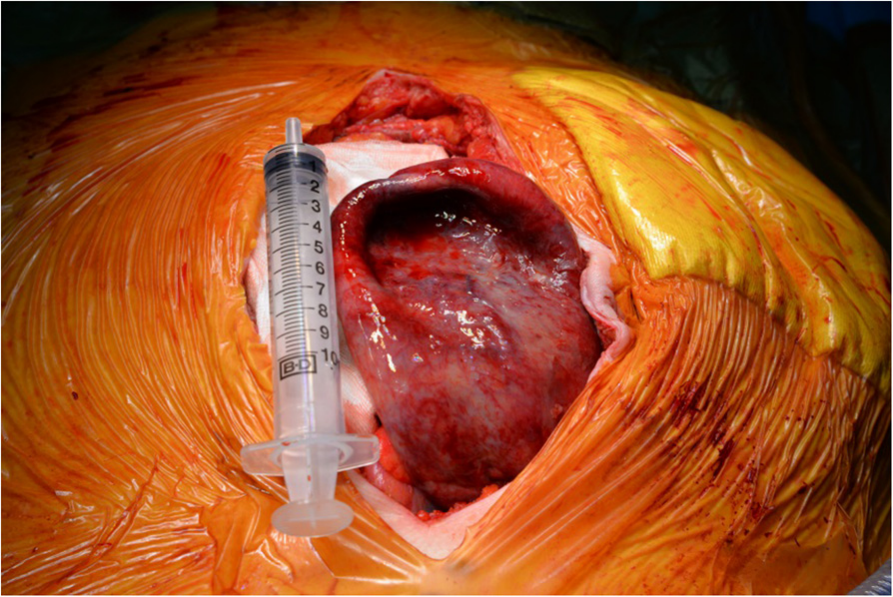
Cholecystitis of the incarcerated gallbladder, 10 mL syringe used for size comparison

At operation on the same day, a urinary catheter was placed in the stoma, and a midline laparotomy was performed. Following dissection and reduction of the midline abdominal hernia, the parastomal defect was defined (Fig. 2). A 14 gauge needle was used to drain the hernia contents of which bile was extracted, confirming the presence of the gallbladder in the hernia (Fig. 3). The gallbladder was then able to be reduced, and noted to be acalculous but thick walled and oedematous (Fig. 4). A cholecystectomy was performed. The large remaining parastomal defect was not closed to not risk the blood supply to the ileal conduit. The patient had an uneventful post-operative course and was discharged on day five. She was well at her one month follow up. Histopathology of the specimen showed chronic cholecystitis without carcinoma.

## Discussion

A gallbladder hernia is a rare event with only a small number of published case reports. The gallbladder has been reported to herniate spontaneously through a ventral defect in the abdominal wall [3, 4], or through a parastomal [5–7], incisional [8–10] or epigastric [11] hernia site. A review of these case reports is able to elicit patterns of presentation and management (Table 1).

**Table 1:** Summary of published case reports of gallbladder hernia indicating patient demographics, hernia type, imaging used for diagnosis and management

Author	Year	Age (year)	Sex	Hernia type	Imaging	Operation	Defect repaired
Goldman et al.	1985	96	F	Epigastric	NA^1^	Laparotomy	Yes
Shirahama et al.	1997	71	F	Incisional	US	Laparotomy	NA
Sirikci et al.	2002	40	F	Incisional	CT	NA	NA
Benzoni et al.	2004	81	F	Incisional	NA	Laparotomy	Yes
Garcia et al.	2005	63	F	Parastomal	CT	Laparotomy	No
St Peter et al.	2005	73	F	Parastomal	CT	Laparotomy	Yes
Rashid et al.	2009	74	F	Parastomal	CT	Laparotomy	Yes, mesh
Paolino et al.	2011	85	F	Spontaneous Ventral	CT	Laparotomy	Yes
Trotta et al.	2013	83	F	Spontaneous Ventral	CT	Laparoscopic	Yes, mesh
Rosenblum et al.	2013	76	M	Parastomal	CT	Laparotomy	NA

^1^NA = not available

As the majority of reported patients were of older age, the mechanism for herniation may be related to the elongation of the gallbladder mesentery with age [12]. This also tends to occurs in females [12]. This demographic pattern also noted in related conditions such as gallbladder torsion [13] and internal gallbladder herniation [14]. As there may also be weakening of the anterior abdominal wall with age, other hernias (such as incisional hernias as observed in this reported case) may also be present.

The presentation of this condition involves an acute or subacute irreducible and often tender abdominal lump. Fever and erythema over the lump may be present with a white cell rise, suggestive of an inflammatory process. Of note, jaundice or abnormal liver function tests are not associated features as there is no biliary obstruction. In addition, vomiting and bowel symptoms are also absent as there is often no associated bowel obstruction.

CT is the imaging modality of choice for diagnosis. A gallbladder hernia may be suspected where there is the absence of the gallbladder from its anatomical position, and a defined, thick walled lesion within the hernia is present which does not contain oral contrast. The gallbladder is often strangulated at its narrow neck, and thus develops cholecystitis and noted to be thick-walled. One case report commented that if gallbladder herniation is suspected, the lump should not be reduced as there may be acute cholecystitis which may result in intra-abdominal contamination [10].

Operative management via a midline laparotomy is preferred. This allows adequate exposure to identify the hernia sac and neck which then permits hernia reduction, cholecystectomy and repair of the defect (where possible). To aid reduction of the gallbladder from its herniated position, decompression may be performed with a wide bore needle. If bile is aspirated, the diagnosis is also confirmed and the decompression may also drain intraluminal infection to prevent contamination.

In the majority of cases, a cholecystectomy was performed to prevent future herniation. In one case, a cholecystectomy was not performed as there was a short time period of presentation, and a non-inflamed gallbladder was observed [5]. In addition to a cholecystectomy, a repair of the hernia defect was often done. This was not performed in this present case report to not risk the blood supply to the ileoconduit. Some reported primary closure of the defect [3, 6, 10] and others performed a mesh closure [4, 7], either of which do not report significant complications. Of note, one case report was able to reduce the hernia and complete the cholecystectomy laparoscopically [4]. This was in a case of spontaneous herniation through the abdominal wall with early identification of the hernia.

Universally, an uncomplicated post-operative recovery was reported. Only one case reported long term follow up, where re-herniation of small bowel through the parastomal defect occurred after 3 years [6].

## Conclusions

In summary, gallbladder herniation is a rare event which may be considered in an elderly patient where there is an acutely tender abdominal lump and CT evidence of the absence of the gallbladder from its anatomical position. The recommended management is an operative approach via a midline laparotomy. Reduction of the hernia may be aided by drainage of the contents. A cholecystectomy should be performed as there is often cholecystitis due to gallbladder incarceration. Repair of the defect should also be performed, unless, in a parastomal herniation, there is risk of compromise the blood supply to the stoma. Laparoscopic repair has been described and may be attempted if access can be gained. There is usually an uneventful post-operative recovery.

## Consent

Written informed consent was obtained from the patient for publication of this case report and any accompanying images. A copy of the written consent is available for review by the Editor of this journal.

## Abbreviations

CT: Computerised tomography
US: Ultrasound
